# An Efficient Finite Element Framework to Assess Flexibility Performances of SMA Self-Expandable Carotid Artery Stents

**DOI:** 10.3390/jfb6030585

**Published:** 2015-07-14

**Authors:** Mauro Ferraro, Ferdinando Auricchio, Elisa Boatti, Giulia Scalet, Michele Conti, Simone Morganti, Alessandro Reali

**Affiliations:** 1 Department of Civil Engineering and Architecture (DICAR), Università di Pavia , Pavia 27100, Italy; E-Mails: auricchio@unipv.it (F.A.); elisa.boatti@unipv.it (E.B.); michele.conti@unipv.it (M.C.); alessandro.reali@unipv.it (A.R.); 2 Laboratoire de Mécanique des Solides, École Polytechnique, Palaiseau 91128, France; E-Mail: scalet@lms.polytechnique.fr; 3 Department of Electrical, Computer and Biomedical Engineering (DIII) , Università di Pavia, Pavia 27100, Italy; E-Mail: simone.morganti@unipv.it; 4 Institute for Advanced Study, Technische Universität München, Lichtenbergstraße 2a, 85748 Garching, Germany

**Keywords:** carotid artery stents, shape memory alloys, finite element analysis, stent flexibility

## Abstract

Computer-based simulations are nowadays widely exploited for the prediction of the mechanical behavior of different biomedical devices. In this aspect, structural finite element analyses (FEA) are currently the preferred computational tool to evaluate the stent response under bending. This work aims at developing a computational framework based on linear and higher order FEA to evaluate the flexibility of self-expandable carotid artery stents. In particular, numerical simulations involving large deformations and inelastic shape memory alloy constitutive modeling are performed, and the results suggest that the employment of higher order FEA allows accurately representing the computational domain and getting a better approximation of the solution with a widely-reduced number of degrees of freedom with respect to linear FEA. Moreover, when buckling phenomena occur, higher order FEA presents a superior capability of reproducing the nonlinear local effects related to buckling phenomena.

## 1. Introduction

Carotid artery stenting (CAS) is a minimally-invasive procedure widely employed for the treatment of atherosclerosis of carotid arteries. In particular, the CAS procedure restores the physiological blood flow by means of the expansion of a metallic endoprosthesis, *i.e*., the stent, which is driven to the target lesion by means of an endoluminal path. Nowadays, CAS is considered a cost-effective alternative to the traditional open surgery approach, leading to minimal hospitalization and reduced social and economic costs [1,2].

Within this framework, it is important to remark that stent delivery is a complex procedure, since the stent needs to accommodate the tortuous path from the incision to the lesion location, avoiding straightening the carotid artery in order to limit vessel injuries. It is immediately clear that stent design plays a crucial role in determining the mechanical properties of the device. The structural requirements of the optimal carotid artery stent are various and, often, contradictory [3,4,5,6]. For this reason, the stents currently available on the market are usually the result of a trade-off among several biomechanical features, experimentally evaluated in order to find a good balance between material properties and design geometry. However, on the one hand, experimental tests are often not applicable due to the high costs of prototype manufacturing, and on the other hand, the realistic working conditions are often difficult to reproduce experimentally.

In this aspect, modern computational methods, typically based on finite element analysis (FEA), are a ubiquitous tool to simulate various clinical procedures for pre-operative planning and to predict the mechanical behavior of a wide range of medical devices [7,8,9,10,11].

Clearly, the reliable application of such computational methods for real-life clinical and industrial problems requires the deep comprehension of the different sources of complexity related to the problem itself. From the material point of view, the majority of modern carotid artery stents is made of NiTiNOL, a nickel-titanium shape memory alloy (SMA) able to elastically recover from strains after stress-induced large deformations [12,13]. SMA behavior is inherently nonlinear, and its macroscopic properties are driven by a complex thermomechanical solid phase transition. In this aspect, modeling such materials requires the development of accurate constitutive laws able to reproduce the major phenomena involved in the material behavior, resorting to a set of parameters possibly limited in number, easy to estimate and underlying a clear physical interpretation. From the numerical point of view, the evaluation of a particular stent feature requires a reliable transposition of the device working conditions into the computational model. This task is not trivial, since real-life problems often include complex nonlinear phenomena, e.g., contact or geometrical instability, that can affect the predicting capabilities of many computational models.

The present paper aims at investigating the capability of dedicated FEA to evaluate an important stent feature, *i.e*., its flexibility (Flexibility is defined as the capability to properly bend in order to accommodate the tortuous vascular structure, and it is considered one of the main features for cardiovascular stents), using advanced constitutive modeling, as well as realistic devices and working conditions. Linear (h-FEA) and higher order (p-FEA) discretizations are adopted to model the 3D stent bending problem in a large deformation regime, corresponding to the cantilever beam bending experiment proposed by Müller-Hülsbeck *et al*. [6]. Linear discretization represents a *de facto* standard for the numerical evaluation of stent mechanical features [14,15,16,17], while the employment of higher order elements allows an accurate representation of the stent geometry combined with better approximation properties with respect to linear FEA. The simulations are performed both using the general purpose solver FEAP [18] and the commercial FEA software Abaqus/Standard for the most computationally-intensive simulations. The SMA model originally proposed by Souza [19] and implemented in the version proposed by Auricchio and Petrini [20] within a large displacement-small strain regime is considered. The results include a performance comparison with respect to the number of degrees of freedom (DOF) and computational times, between h-FEA and p-FEA. Moreover, we highlight the capability of the two methods to reproduce the nonlinear local effects due to geometrical instability.

The paper is structured as follows: In Section 2, we describe the proposed computational framework, including SMA constitutive relations, stent geometrical modeling and the implemented analysis setup. In Section 3, we present and discuss some numerical results for both h- and p-FEA. This section is structured in order to highlight not only a general comparison in terms of the number of degrees of freedom, but also a focus on local nonlinear effects. Finally, in Section 4, we summarize our findings.

## 2. Materials and Methods

In this section, the SMA constitutive model used in the present study is described. In particular, the time-continuous and time-discrete frameworks, as well as the adopted large displacement-small strain implementation are detailed. Subsequently, the computational framework to obtain both linear and p-FEA stent models is described. The resulting model is then integrated within an analysis setup simulating an experimental stent bending test.

### 2.1. Souza-Auricchio Model: Time-Continuous Framework

Following the works by Souza *et al*. [19] and Auricchio and Petrini [20], we adopt a 3D constitutive model developed within the framework of phenomenological continuum thermomechanics and able to describe the main SMA macroscopic behaviors. The assumed control variables are the total strain *ε* and the absolute temperature *T*, while the transformation strain $e^{tr}$ is taken as an internal variable. The transformation strain satisfies the constraint:

(1) $$∥e^{tr}∥\leq \varepsilon_{L}$$

where $\varepsilon_{L}$ is a material parameter corresponding to the maximum transformation strain reached at the end of the phase transformation during an uniaxial test.

The Helmoltz free energy density function $\text{Ψ}=\text{Ψ}(\varepsilon ,T,e^{tr})$ is used as the thermodynamic potential as follows

(2) $$\text{Ψ}=\frac{1}{2}\;\kappa \;\theta^{2}+G\;∥e-e^{tr}{∥}^{2}+\tau_{M}\;∥e^{tr}∥+\frac{1}{2}\;h\;{∥e^{tr}∥}^{2}+I_{\varepsilon_{L}}\left(e^{tr}\right)$$

*θ* and *e* being the volumetric and deviatoric part of the total strain *ε*, respectively; $\tau_{M}=\beta \langle T-T^{*}\rangle$, where *β* is a positive parameter related to the dependence of the critical stress on temperature, $T^{*}$ a reference temperature and 〈·〉 indicates the positive part function; *κ* and *G* are the bulk and shear modulus, respectively; *h* defines the phase transformation hardening. The indicator function:

(3) $$I_{\varepsilon_{L}}\left(e^{tr}\right)=\left\{\begin{array}{cc} 0 & \mathrm{if}\;∥e^{tr}∥\leq \varepsilon_{L}\\ +\infty & \mathrm{otherwise} \end{array}\right.$$

is introduced to satisfy the transformation strain constraint of Equation (1).

Following standard arguments [21], the constitutive equations can be differentiated:

(4) $$\left\{\begin{array}{c} p=\frac{\partial \text{Ψ}}{\partial \theta }=\kappa \theta \\ s=\frac{\partial \text{Ψ}}{\partial e}=2G\left(e-e^{tr}\right)\\ X=-\frac{\partial \text{Ψ}}{\partial e^{tr}}=s-\tau_{M}\frac{e^{tr}}{∥e^{tr}∥}-he^{tr}-\gamma \frac{e^{tr}}{∥e^{tr}∥} \end{array}\right.$$

where *p* and *s* are the volumetric and deviatoric part of the stress ***σ***, respectively, and ***X*** is the thermodynamic stress-like quantity associated with the transformation strain $e^{tr}$. The variable *γ* results from the indicator function subdifferential $\partial I_{\varepsilon_{L}}\left(e^{tr}\right)$, and it is defined as follows:

(5) $$\gamma =\left\{\begin{array}{cc} 0 & \mathrm{if}\;∥e^{tr}∥<\varepsilon_{L}\\ \geq 0 & \mathrm{if}\;∥e^{tr}∥=\varepsilon_{L} \end{array}\right.$$

yielding $\partial I_{\varepsilon_{L}}\left(e^{tr}\right)=\gamma e^{tr}/∥e^{tr}∥$.

A classical Mises-type limit function F=F(X) is introduced as:

(6) $$F=∥X∥-R_{Y}$$

where $R_{Y}$ is a positive material parameter corresponding to the elastic radius in the deviatoric space. The evolution equation for the internal variable takes the form:

(7) $${\dot{e}}^{tr}=\dot{\lambda }\frac{\partial F}{\partial X}=\dot{\lambda }\frac{X}{∥X∥}$$

where $\dot{\lambda }$ is the non-negative consistency parameter. The model is finally completed by the classical Kuhn–Tucker conditions:

(8) $$\dot{\lambda }\geq 0,\;\;F\leq 0,\;\;\dot{\lambda }F=0$$

### 2.2. Souza-Auricchio Model: Time-Discrete Framework

Starting from the material state at time $t_{n}$, identified by the quantities $\left(e_{n}^{tr},\lambda_{n},\gamma_{n}\right)$, we admit global guess values of the total strain *ε* and temperature *T* at time $t_{n+1}$. A return map procedure, based on the elastic predictor/inelastic corrector scheme [22], is adopted to compute the stress and the other variables at the current time $t_{n+1}$. For the sake of notational simplicity, we omit the subscript n+1 for all of the variables computed at the current time $t_{n+1}$ and adopt subscript *n* for the variables computed at time $t_{n}$. The implicit backward Euler integration scheme is employed within the evolution equation in the following form:

(9) $$e^{tr}=e_{n}^{tr}+\Delta \lambda \frac{X}{∥X∥}$$

where $\Delta \lambda =\int_{t_{n}}^{t_{n+1}}\dot{\lambda }\;\;dt$ is the time-integrated consistency parameter. The algorithm consists of evaluating an elastic trial state (denoted with subscript *TR*) in which the internal variables remain constant, *i.e*.,

(10) $$\left\{\begin{array}{c} \Delta \lambda_{TR}=0\\ e_{TR}^{tr}=e_{n}^{tr}\\ \gamma_{TR}=0 \end{array}\right.$$

from which:

(11) $$\left\{\begin{array}{c} s_{TR}=2G\left(e-e_{TR}^{tr}\right)\\ X_{TR}=s_{TR}-\tau_{M}\frac{e_{TR}^{tr}}{\bar{∥e_{TR}^{tr}∥}}-he_{TR}^{tr} \end{array}\right.$$

Then, the limit function (see Equation (6)) is computed to verify the admissibility of the trial state. If the trial state is admissible, the step is elastic; otherwise, the step is inelastic, and the transformation strain has to be updated through the time-discrete evolution equation (see Equation (9)). We perform the inelastic step by solving the following non-linear system with a Newton–Raphson method:

(12) $$\left\{\begin{array}{c} e^{tr}-e_{n}^{tr}-\Delta \lambda \frac{X}{∥X∥}=0\\ ∥X∥-R_{Y}=0 \end{array}\right.$$

If the above solution is not admissible (*i.e*., if constraint (1) is not verified), a further inelastic step is performed for saturated conditions, and the following non-linear system is solved with a Newton–Raphson method:

(13) $$\left\{\begin{array}{c} e^{tr}-e_{n}^{tr}-\Delta \lambda \frac{X}{∥X∥}=0\\ ∥X∥-R_{Y}=0\\ ∥e^{tr}∥-\varepsilon_{L}=0 \end{array}\right.$$

It is important to remark that the ratio $e^{tr}/∥e^{tr}∥$ is undefined when $e^{tr}$ is null. Therefore, the euclidean norm $∥e^{tr}∥$ is opportunely replaced with the following regularized expression:

(14) $$\bar{∥e^{tr}∥}=\sqrt{∥e^{tr}{∥}^{2}+\delta }-\sqrt{\delta }$$

where *δ* is a user-defined positive regularization parameter (∼10^−8^). We remark that the implicit implementation requires also the formulation of the consistent tangent matrix ℂ (see [20] for the corresponding expression).

### 2.3. Souza-Auricchio Model: Numerical Implementation

The Souza-Auricchio model is implemented as a user material subroutine (UMAT) for both FEAP and Abaqus/Standard Version 6.11 (Dassault Systémes, Johnson, RI, USA). The hypothesis of large displacements and rotations, but small strains (as typically induced in many biomedical applications [17]), is assumed. In particular, starting from the deformation gradient **F** provided by the solver, we compute the Green–Lagrange strain tensor as $\text{E}=\frac{1}{2}\left({\text{F}}^{T}\text{F}-\text{I}\right)$. Then, we use the following additive decomposition of the strain tensor **E**:

(15) $$\text{E}=\text{e}+\frac{1}{3}\theta \text{I}$$

where:

(16) $$\theta =tr\left(\text{E}\right)\;\text{e}=\text{E}-\frac{1}{3}\theta \text{I}$$

The quantities **e** and *θ*, coupled with the temperature *T* and the internal variable ${\text{e}}^{tr}$, are used to compute the second Piola–Kirchhoff stress tensor $\text{S}=\text{s}+\frac{1}{3}p\text{I}$ and the consistent tangent matrix ℂ (see Equation (4) and Section 2.2). Both **S** and ℂ are expressed in terms of the reference configuration, and a push forward procedure needs to be applied in order to obtain the Cauchy stress tensor *σ* and the consistent tangent matrix **c** in terms of the current configuration. In particular, we have:

(17) $$\sigma =\frac{1}{J}{\text{FSF}}^{T}$$

(18) $$\text{c}=\frac{1}{J}\phi_{*}\left[\text{ℂ}\right]$$

where $\phi_{*}$ refers to the compact notation for the push forward operation on fourth order tensors, as described in [23].

### 2.4. Stent Model

**Figure 1:**
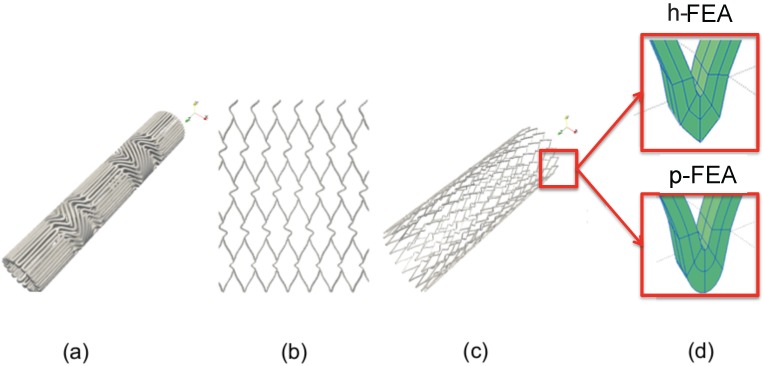
Stent model generation: (**a**) detail of a high resolution micro-CT performed on the real stents within the delivery system; (**b**) planar CAD geometry reproducing the stent design pattern; (**c**) 3D CAD stent model; (**d**) h- (top) and p- (bottom) finite element analysis (FEA) mesh generation.

A novel computational framework to interface the CAD software Rhinoceros v. 4.0 SR8 (McNeel and Associated, Seattle, WA, USA) with the general purpose solver FEAP is presented. The stent model used in the present work resembles a commercially available stent used in clinical practice, *i.e*., a XACT Carotid Stent (Abbott, IL, USA). This device is characterized by a closed-cell design, since all of the junctions between different rings are connected. This feature strongly influences many biomechanical outcomes, e.g., vessel scaffolding, adaptability and, also, flexibility. We consider a straight configuration having a 9-mm reference internal diameter, a 0.18-mm thickness and a 30-mm length. Since no data are available from the manufacturer, the main geometrical features of such devices are derived from high-resolution micro-CT scans (*cf*. Figure 1a) of the crimped stent in the delivery system [7]. The FEA stent model is generated through the following steps:

- The geometrical features derived by the micro-CT scans are elaborated using a parametrical model, in order to obtain a geometrical description corresponding to the unfold stent in open configuration.
- A planar CAD geometry (see Figure 1b) is generated. Subsequently, a 2D CAD NURBS surface for the whole stent is created.
- The CAD surface structure is extruded and rolled by means of an in-house MATLAB code (The MathWorks Inc. Natick, MA, USA) leading to the final stent in open configuration, as depicted in Figure 1c.
- The 3D trivariate CAD data are processed in order to obtain both h- and p-FEA meshes. At last, the FEA mesh is exported in a suitable format for the solver FEAP.

For the h-FEA, we employ traditional trilinear brick elements with full integration, while for the p-FEA, we employ cubic-cubic-quadratic brick elements (for circumferential, longitudinal and thickness directions, respectively) with full integration. In particular, the p-FEA polynomial orders are obtained by construction of the 2D NURBS surface for circumferential and longitudinal directions, while the thickness polynomial order, linear by construction, has been raised to quadratic in order to have a fully high order p-FEA mesh. Both refinement techniques are implemented using an in-house MATLAB code based on the NURBS toolbox [24,25], a set of routines implementing the algorithms included in [26]. In particular, the p-FEA mesh is recovered by iterative knot insertion on a highly regular NURBS mesh.

### 2.5. Analysis Setup

**Figure 2:**
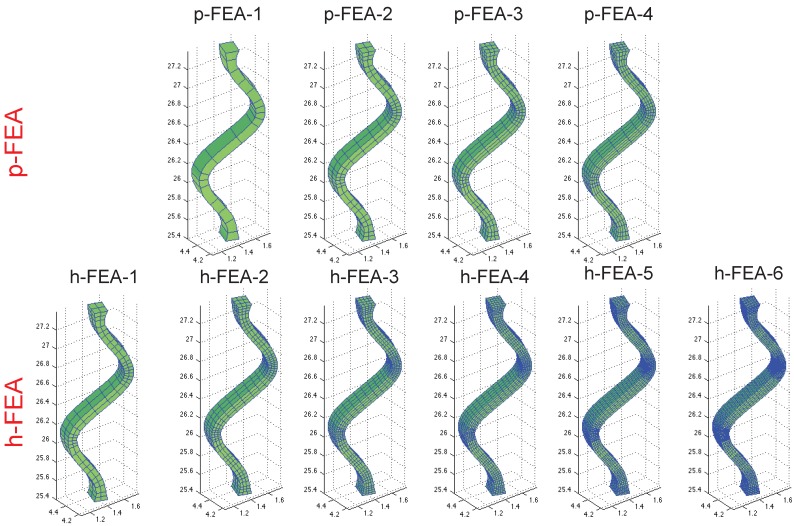
Stent refinement levels: top p-FEA; bottom h-FEA.

Following the computational framework proposed by Auricchio *et al*. [27], the flexibility test is simulated through a displacement-based analysis in the large deformation regime. A displacement of 11 mm along the *Y* direction is imposed for all of the nodes referring to the distal extremity of the stent, while the proximal one is clamped. We consider the resultant reaction force at the distal extremity of the device as a reference quantity to evaluate the capability of both h- and p-FEA to correctly reproduce the stent bending, as also used in the experimental setup proposed by Müller-Hülsbeck *et al*. [6]. In particular, the resultant is obtained as the sum of the reaction force contributions at the distal extremity of the stent. The Souza-Auricchio model constitutive parameters are obtained from the literature [27]. We use 6 and 4 refined meshes for h- and p-FEA, respectively (the different refinement levels are shown in Figure 2). As introduced in Section 1, h-FEA-5 and h-FEA-6 simulations are computationally intensive, and thus, for efficiency reasons, they are performed using the Abaqus/Standard solver (Both h-FEA and p-FEA simulations are performed on an Intel Xeon E5-4620 at 2.20 GHz workstation with 252 GB RAM). The description of all analyses in terms of numbers of degrees of freedom (DOF) and polynomial degrees can be found in Table 1.

## 3. Results and Discussion

**Figure 3:**
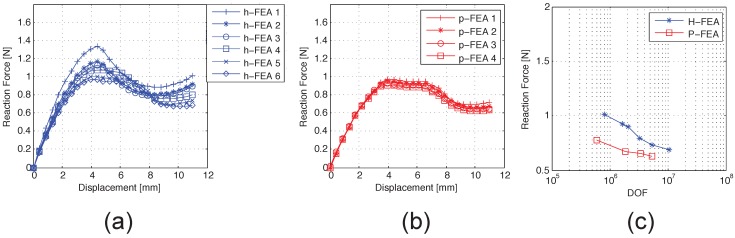
Force-displacement diagrams for shape memory alloy (SMA) stent bending: (**a**) FEA; (**b**) p-FEA; (**c**) reaction force convergence plot.

The present work aims at investigating the potential of dedicated FEA in simulating the stent flexibility behavior comparing the performance of h- and p-FEA discretizations. As previously indicated, we consider the resultant reaction force at the distal extremity of the stent as a reference quantity to evaluate the performance with respect to the number of DOF. In particular, the force-displacement curves for h-FEA and and p-FEA simulations are depicted and compared in Figure 3a,b. Moreover, reaction force convergence plots with respect to the DOF number for both methods are reported in Figure 3c. Finally, the data concerning reaction force values and numerical errors, evaluated with respect to the results from the most refined p-FEA analysis (p-FEA-4) are reported in Table 1.

**Table 1:** Stent bending analyses: the relative errors are evaluated with respect to the finest P-FEA simulation, labeled as P-FEA-4. The symbols p, q and r indicate the circumferential, longitudinal and thickness polynomial orders of the FEA meshes, respectively.

Mesh Label	DOF	Order			Reaction Force (N)		Critical Load (N)	
		**p**	**q**	**r**	**Value**	**Error**	**Value**	**Error**
FEA-1	606,276	1	1	1	1.0102	60.21%	1.3354	47.76 %
FEA-2	1,635,960	1	1	1	0.91935	51.85%	1.1671	29.14 %
FEA-3	2,118,096	1	1	1	0.89916	42.60 %	1.1300	25.03 %
FEA-4	3,246,480	1	1	1	0.79611	26.25 %	1.0754	18.99 %
FEA-5	5,281,740	1	1	1	0.73349	16.32 %	0.99991	10.64 %
FEA-6	10,622,016	1	1	1	0.68897	9.26 %	0.97022	7.35 %
p-FEA-1	598,212	3	3	2	0.7725	22.51 %	1.0342	14.31 %
p-FEA-2	1,844,820	3	3	2	0.6732	6.76%	0.9544	5.49 %
p-FEA-3	3,469,668	3	3	2	0.6480	2.76 %	0.91242	0.8 %
p-FEA-4	5,269,642	3	3	2	0.63054	–	0.90473	–

The results show that, as expected, p-FEA presents a better performance with respect to h-FEA on a per degree of freedom basis, with a gain of about one order of magnitude in terms of DOF number. In particular, except the coarsest mesh p-FEA-1, which presents some unphysical local buckling, all of the p-FEA meshes show a numerical error below 7%. As an example, we fix the DOF number in the range of $2\cdot 10^{6}$, corresponding to the meshes p-FEA-2 and h-FEA-3, respectively. In particular, p-FEA-2 shows a numerical error of 6.76% with respect to the converged solution p-FEA-4, while h-FEA-3 shows a relative error of 42.60%. Moreover, the most refined h-FEA, comprising over ten million DOF, still shows an error of 9.26%.

As introduced previously, we now focus on the influence of stent design, with particular care for kink formation and the buckling phenomenon, which often appear when a closed-cell stent is considered. Kink resistance is an important feature of stent devices [28]. When strains locally increase beyond the critical value, the buckling phenomenon occurs, inducing high stresses. This phenomenon can be very dangerous for device performance, since it can lead to the reduction of the fatigue life and implant failure. In particular, closed-cell designs show reduced adaptability and are prone to kinking [7].

**Figure 4:**
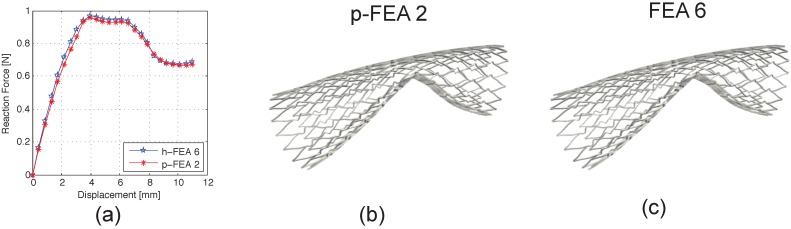
FEA *versus* p-FEA comparison: (**a**) force displacement curves obtained using the finest FEA mesh (FEA-8) and a coarse p-FEA mesh (p-FEA-2); (**b**) p-FEA-2 deformed configuration; (**c**) FEA-8 deformed configuration.

Our results confirm this statement, also in accordance with experimental results [6] (Figure 4b,c). From a computational viewpoint, in Figure 3, it is possible to observe that, while p-FEA presents the same deformation pattern for all considered refinements, h-FEA shows different behaviors with different refinements, which are related to some spurious stress concentrations that lead to an erroneous reproduction of the buckling deformation path. In particular, the p-FEA deformation pattern shows two stages of local buckling regardless of the refinement level (see Figure 3b). On the other hand, h-FEA is not able to catch this local behavior until a high number of DOF is included (see Figure 3a). This phenomenon can be better appreciated in Figure 4a. This aspect has a great influence on the capability of accurately reproducing the value of the critical load (see Table 1).

### 3.1. Computational Times

Even though a fair efficiency comparison of h-FEA and p-FEA stent bending simulations requires the employment of the same software package, it is interesting to provide the computational times for a given level of accuracy. In particular, the computational time coming from two simulations with comparable levels of accuracy, *i.e*., p-FEA-2 mesh and h-FEA-6 mesh, are reported in Table 2. In this case, while the p-FEA-2 simulation is performed with FEAP and one CPU, the h-FEA-6 simulation is performed with Abaqus/Standard with eight CPUs, for the reason of the motivations previously mentioned. However, it is interesting to remark that, from a qualitative point of view, p-FEA shows comparable times with respect to h-FEA, despite the difference in the number of used CPUs.

**Table 2:** Computational times for p-FEA and h-FEA.

Mesh Label	DOF	No. of CPUs	Solver	Total Analysis Time
p-FEA-2	1,844,820	1	FEAP	27 h 15 min
FEA-6	10,622,016	8	Abaqus/Standard	26 h 23 min

## 4. Conclusions

In the present study, we developed a numerical bench test able to integrate stent FEA models and inelastic SMA constitutive modeling, in order to evaluate the flexibility of self-expandable carotid artery stents. We implemented a computational framework able to employ both h-FEA and p-FEA, with the ultimate goal to compare the numerical performance of high order basis with respect to the standard linear basis. Our results suggest that the employment of p-FEA allows one to accurately represent the computational domain and to obtain a better approximation of the solution with a reduced number of DOF with respect to h-FEA. Moreover, when buckling phenomena occur and geometrical accuracy plays an important role, p-FEA presents a superior capability to reproduce the nonlinear local effects. The full exploitation of the present framework within the stent development requires different advancements, including the coupling of flexibility test with imaging techniques of real devices, the calibration of the SMA model parameters, the introduction of realistic vessel models and the implementation of a robust contact driver to simulate the stent-vessel interaction. Moreover, the cutting-edge research on stents requires the use of explicit dynamics solvers, able to get rid of the high nonlinearity of such simulations. In this aspect, it is well known in the literature that p-FEA are not able to correctly reproduce a wide range of the frequency spectrum, while the use of linear elements mitigate this adverse effect [29,30]. Some recent results [31] demonstrate that the introduction of more sophisticated basis, *i.e*., NURBS-based isogeometric analysis [32], characterized by high order and high regularity basis functions, provides excellent results also for explicit dynamics simulations [33].
